# Supplementary material for: 1,25-Dihydroxyvitamin D Enhances the Regenerative Function of Lgr5+ Intestinal Stem Cells In Vitro and In Vivo
Source: Cells. 2024 Aug 31;13(17):1465. doi: 10.3390/cells13171465 (PMC11394149; doi:10.3390/cells13171465)
Supplement: Supplementary file 1 [file cells-13-01465-s001.zip › Supplementary Materials.pdf]

1,25-Dihydroxyvitamin D Enhances the Regenerative Function of $Lgr5^+$ Intestinal Stem Cells in Vitro and in Vivo
(Supplemental Materials)

Nisar Ali Shaikh [1], Chenfan Liu [1,2], Yue Yin [1], David J. Baylink [3,4] and Xiaolei Tang [1,3,4,*]

[1]Department of Veterinary Biomedical Sciences, College of Veterinary Medicine, Long Island University, Brookville, NY 11548, USA; shaikh.nisarali@liu.edu (N.A.S.); bpflcf@outlook.com (C.L.); yinyuecindy@gmail.com (Y.Y.)
[2]Shandong Public Health Clinical Center, Shandong University, Shandong 250013, China
[3]Division of Regenerative Medicine, Department of Medicine, School of Medicine, Loma Linda University, Loma Linda, CA 92354, USA; dbaylink@llu.edu
[4]Department of Basic Sciences, School of Medicine, Loma Linda University, Loma Linda, CA 92354, USA
*Correspondence: xiaolei.tang@liu.edu

Table S1:

Real-time RT-qPCR primers				
S.No	Gene	Primer	Sequence (5' → 3')	
	Mouse			
1.	GAPDH	Forward	TGGCCTTCCGTGTTCCTAC	
		Reverse	TGCGACTTCAACAGCAACTC	
2.	Ki67	Forward	GAGGAGAAACGCCAACCAAGAG	
		Reverse	TTTGTCCTCGGTGGCGTTATCC	
3.	Bcl2	Forward	CCTGTGGATGACTGAGTACCTG	
		Reverse	AGCCAGGAGAAATCAAACAGAGG	
4.	Caspase3	Forward	GGAGTCTGACTGGAAAGCCGAA	
		Reverse	CTTCTGGCAAGCCATCTCCTCA	
5.	Lgr5	Forward	AGAGCCTGATACCATCTGCAAAC	
		Reverse	TGAAGGTCGTCCACACTGTTGC	
6.	Sox9	Forward	CACACGTCAAGCGACCCATGAA	
		Reverse	TCTTCTCGCTCTCGTTCAGCAG	
7.	Smoc2	Forward	GGAAGGAGCAGGGAAAGCAGATGAT	
		Reverse	TGGGCTGCTTGGCTTCCTCAAG	
8.	Ascl2	Forward	CCTCTCTCGGACCCTCTCTCAG	
		Reverse	CAGTCAAGGTGTGCTTCCATGC	
9.	Alpi	Forward	CCAGCAGTAACTCACCTCATGG	
		Reverse	GAAGCCTTGTGGATTCCTGCTG	
10.	Chga	Forward	AGAACCAGAGCCCTGATGCCAA	
		Reverse	CTCTGTGGTTGCCTCAAAGCCA	
11.	Muc2	Forward	GCTGACGAGTGGTTGGTGAATG	
		Reverse	GATGAGGTGGCAGACAGGAGAC	
12.	Lyz1	Forward	TACAACCGTGGAGACCGAAGCA	
		Reverse	TGGCTGCAGTGATGTCATCCTG	
13.	Vdr	Forward	GCTCAAACGCTGCGTGGACATT	
		Reverse	GGATGGCGATAATGTGCTGTTGC	
14.	Cyp27b1	Forward	TTCGGCTTTGGCAAACGGAGCT	
		Reverse	GGCTTGATAGGAAGAGCACCTG	
15.	Cyp24a1	Forward	GCTCCTTCAAAAGGACACAGAGG	
		Reverse	CGCTTGCCACACTTTGGTGTTG	
	Human			
16.	GAPDH	Forward	GTCTCCTCTGACTTCAACAGCG	
		Reverse	ACCACCCTGTTGCTGTAGCCAA	
17.	Cyp27b1	Forward	CTCCACTCAGAGATCACAGCTG	
		Reverse	GGACACGAGAATTTCCAGGTACC	

Genotyping PCR primers

1. B6.129P2-*Lgr5*$^{tm1(cre/ERT2)Cle}$/J

PRIMER	SEQUENCE 5' → 3'	PRIMER TYPE	NOTE
**15020**	CTG AAC TTG TGG CCG TTT AC	Mutant Reverse	
**26840**	GTC TGG TCA GAA TGC CCT TG	Wild type Reverse	
**8060**	CTG CTC TCT GCT CCC AGT CT	Common	

2. ***Gt(ROSA)26Sor***$^{tm9(CAG\text{-}tdTomato)Hze}$

PRIMER	SEQUENCE 5' → 3'	PRIMER TYPE	NOTE
**oIMR9020**	AAG GGA GCT GCA GTG GAG TA	Wild type Forward	
**oIMR9021**	CCG AAA ATC TGT GGG AAG TC	Wild type Reverse	
**oIMR9103**	GGC ATT AAA GCA GCG TAT CC	Mutant Reverse	WPRE
**oIMR9105**	CTG TTC CTG TAC GGC ATG G	Mutant Forward	tdTomato

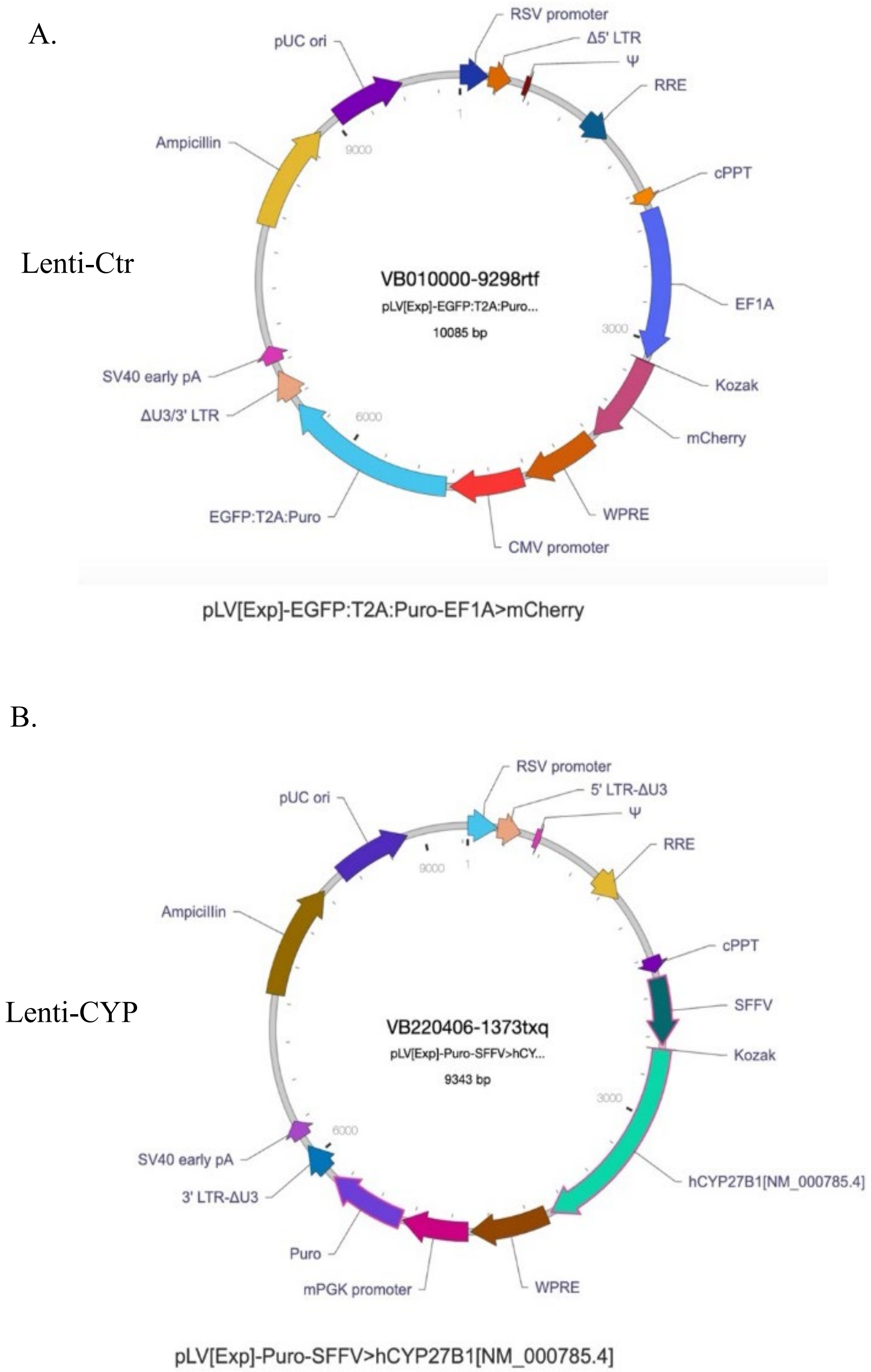

Figure S1: Lentivirus vectors used in this study. ***A)*** The control lentiviral vector, *i.e.*, lenti-Ctr, was the control empty vector containing the GFP and puromycin-resistant gene ***B)*** Lenti-hCYP was the vector that contained the human CYP27B1 gene (hCYP27B1) driven by the SFFV promoter and the puromycin resistant gene.

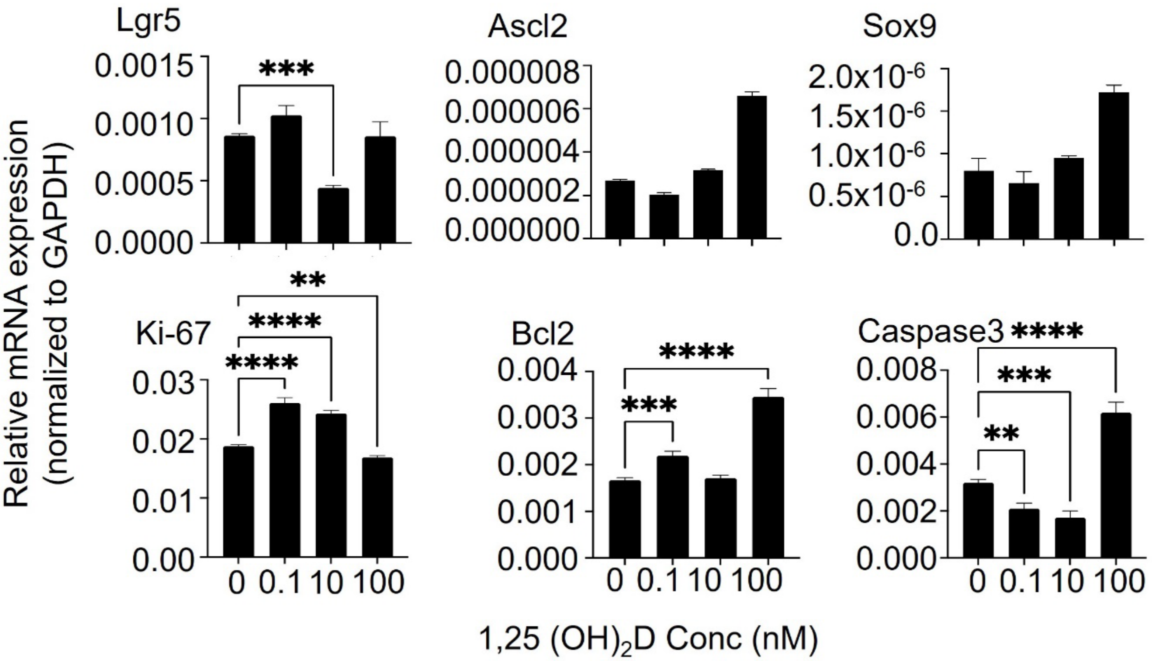

Figure S2. 1,25(OH)$_2$D does not suppress the stemness of Lgr5$^+$ ISCs in the presence of valproic acid and CHIR99021. The Lgr5$^+$ ISC lines were cultured in the presence of the chemical inhibitors and treated with different 1,25(OH)$_2$D concentrations (0, 0.1, 10, and 100 nM) for one week. The cells were analyzed for the markers of stemness (Lgr5, Ascl2, and Sox9), proliferation (Ki-67), and apoptosis (Bcl2 and caspase 3) by real-time RT-qPCR. Data were normalized to the housekeeping gene GAPDH. Bars represent mean ± standard error of the mean (SEM) (n=3). **P<0.01, ***P<0.001, ****P<0.0001. Ordinary one-way ANOVA.

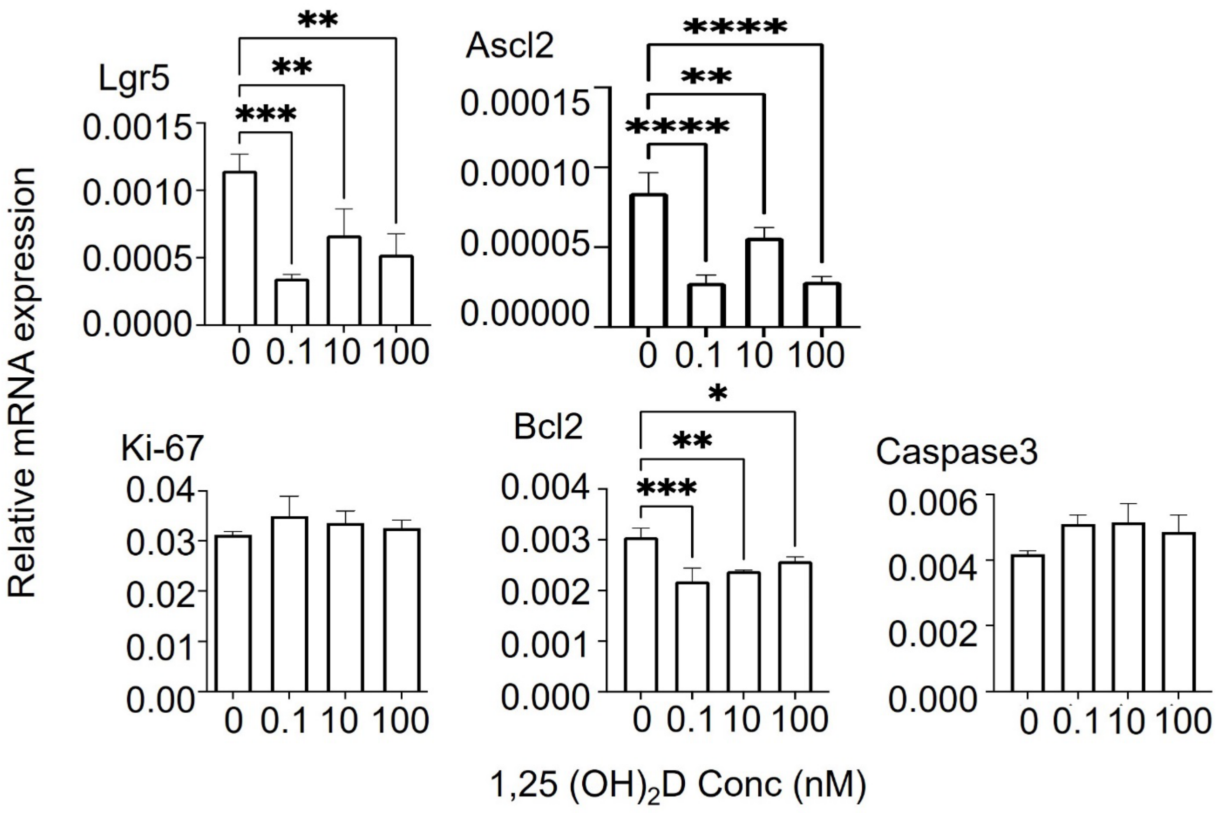

Figure S3. 1,25(OH)$_2$D downregulates the expressions of the markers for stemness but does not suppress proliferation in the absence of valproic acid and CHIR99021. The Lgr5$^+$ ISC lines were cultured in the absence of the chemical inhibitors to initiate differentiation and, at the same time, treated with different 1,25(OH)$_2$D concentrations (0, 0.1, 10, and 100 nM) for one week. The cells were analyzed for the markers of stemness (Lgr5, Ascl2, and Sox9), proliferation (Ki-67), and apoptosis (Bcl2 and caspase 3) by real-time RT-qPCR. Data were normalized to the housekeeping gene GAPDH. Bar represents the mean ± standard error of the mean (SEM) (n=3). *P<0.05, **P<0.01, ***P<0.001, ****P<0.0001. Ordinary one-way ANOVA.

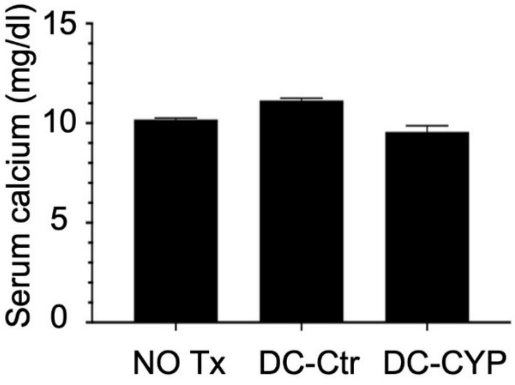

Figure S4. Peritoneal injection of DC-CYP cells does not cause hypercalcemia. Lgr5GFP-AI mice were generated by breeding the cre-inducible Lgr5-EGFP-IRES-creERT2 (Lgr5-GFP) mice with the cre-reporter B6.Cg-Gt(ROSA)26Sort m9(CAG-tdTomato)Hze/J (Ai9 or Ai) mice. The mice were intraperitoneally administered with tamoxifen (75 mg/kg body weight). On the second day, the mice received 2 x 10^6 cells/mouse of either DC-Ctr or DC-CYP cells. In addition, one group of mice that did not receive treatment (NO Tx) was included as a control. Five days after the cell treatment, sera were analyzed for calcium concentrations. Data from one of the two independent experiments are shown as means ± SEM (n = 3).

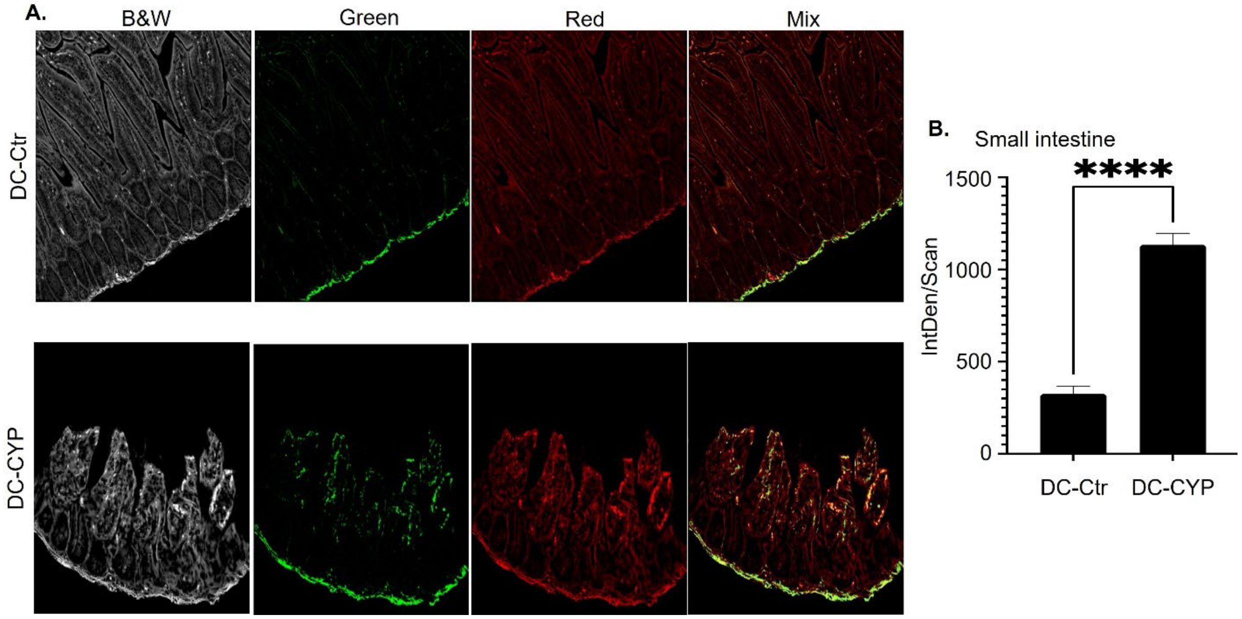

Figure S5. Peritoneal injection of DC-CYP cells enhanced Lgr5⁺ ISC differentiation *in vivo*. Lgr5GFP-AI mice were generated by breeding the cre-inducible Lgr5-EGFP-IRES-creERT2 (Lgr5-GFP) mice with the cre-reporter B6.Cg-Gt(ROSA)26Sor tm9(CAG-tdTomato)Hze/J (Ai9 or Ai) mice. The mice were intraperitoneally administered with tamoxifen (75 mg/kg body weight). On the second day, the mice received 2 x 10^6 cells/mouse of either DC-Ctr or DC-CYP cells. Five days after the cell treatment, small intestines were processed for cry sectioning and analyzed by fluorescence microscope. ***A)*** Representative small intestine images from mice treated with DC-Ctr or DC-CYP cells. The green color represents GFP, and the red color represents tdTomato. ***B)*** Data were intensities of tdTomato$^+$ cells (red, crypts were excluded) from three randomly chosen microscopic fields per scan and quantified by Fiji software. Statistical analysis was done by two-way ANOVA.
